# Understanding the clinical and molecular epidemiological characteristics of carbapenem-resistant *Acinetobacter baumannii* infections within intensive care units of three teaching hospitals

**DOI:** 10.1186/s12941-024-00766-4

**Published:** 2025-01-13

**Authors:** Pengyu Zhang, Jingchen Hao, Yafen Zhang, Junfeng Su, Guozhuang Sun, Jun Xie, Jian Hu, Guocai Li

**Affiliations:** 1 Department of Microbiology, Medical College, Yangzhou University, Yangzhou, 225001 China; 2 Jiangsu Key Laboratory of Zoonosis/Jiangsu Co-Innovation Center for Prevention and Control of Important Animal Infectious Diseases and Zoonoses, Yangzhou University, Yangzhou, 225009 China; 3 Jiangsu Key Laboratory of Integrated Traditional Chinese and Western Medicine for Prevention and Treatment of Senile Diseases, Yangzhou, 225009 China; 4 Department of Laboratory Medicine, Affiliated Hospital of Yangzhou University, Yangzhou, 225001 PR China; 5 Department of Laboratory Medicine, Xuyi County People’s Hospital / Clinical College, Yangzhou University, Yangzhou, 211799 China; 6 Department of Laboratory Medicine, Guangling College / Clinical College, Yixing Hospital of Traditional Chinese Medicine, Yangzhou University, Yangzhou, 214200 China

## Abstract

**Background:**

Carbapenem-resistant *Acinetobacter baumannii* (CRAB) is recognized as a common clinical conditional pathogen with *bla*_OXA−23_ gene-mediated multidrug-resistance that is a significant threat to public health safety. Timely and effective infection control measures are needed to prevent their spread.

**Methods:**

We conducted a retrospective study of CRAB patients at three teaching hospitals from 2019 to 2022. We identified bacterial isolates, collected clinical data, and performed antimicrobial susceptibility testing. Genome characteristics of isolates were investigated by whole genome sequencing. Multilocus sequence typing and phylogenetic trees were used to assess the genetic similarity of isolates. Acquired antimicrobial resistance genes and virulence factors carried in the isolated group genome were analyzed by ResFinder, PubMLST and VFDB. Sequence alignment was used to analyze genetic environment around *bla*_OXA−23_. Phylogenetic tree was constructed to analyze the genetic relationship of isolates.

**Results:**

A total of 92 non-repetitive CRAB isolates were collected, with sputum samples accounting for the majority (94.57%, *n* = 87) of samples. These were distributed into ST2, with ST2 identified to have the highest prevalence of infection, accounting for 99.99% (*n* = 91) of all isolates. The major resistance genes identified were *bla*_OXA−23_, *bla*_OXA−66_, *bla*_OXA−51_, and *bla*_ADC_. Also, 92 CRAB strains showed high levels of resistance to common clinical antibiotics, but not minocycline. Meanwhile, most of the isolates carried virulence genes such as various *ompA*, *csuA*, *csuB*, *csuC*, *csuD*, *abaI*, *abaR*, *lpxC*, *lpxA*, and *bmfRS*. Single nucleotide polymorphism (SNP) analyses further indicated that the bacterial genome was progressively polymorphic with time. We analyzed the environment of the *bla*_OXA−23_ gene and found that CRAB accumulated in the context of prominent environmental antibiotic exposure and had longer survival times in the antibiotic environment, resulting in the tendency of bacteria to develop greater antibiotic resistance.

**Conclusions:**

We find that CRAB is prevalent within the ICU and is progressively resistant to antibiotics over time. Enhanced clinical understanding and timely management of CRAB infections will be crucial to minimize or even eliminate the spread of CRAB within the ICU setting.

**Supplementary Information:**

The online version contains supplementary material available at 10.1186/s12941-024-00766-4.

## Introduction

Bacterial infections are significant causes of deaths in patients in ICU, and increasing antibiotic resistance in bacteria has led to enhanced risks of such nosocomial complications in primary care and surgery. In addition, the growing emergence of multidrug resistant (MDR) microorganisms is evolving to become an increasing burden on the cost of medical care of many countries. The most prominent species of MDR bacteria currently evading antimicrobial therapy that is also spreading worldwide are known as ESKAPE microorganisms: *Enterococcus faecalis*, *Staphylococcus aureus*, *Klebsiella pneumoniae*, *Acinetobacter baumannii*, *Pseudomonas aeruginosa* and *Enterobacter spp*. Notably, among these, the number of infections caused by Gram-negative *Acinetobacter baumannii* is increasing in incidence worldwide every year [1]. Moreover, the World Health Organization (WHO) has included carbapenem-resistant *A. baumannii* as a strain that poses the greatest threat to human health and is recommending the prioritization of research to develop new antimicrobial therapies to treat such infections (https://www.who.int/).

*Acinetobacter baumannii* is a pathogen that causes ventilator-associated pneumonia, catheter-associated infections, and skin and urinary tract infections. Surveillance data from China shows that more than 50% of *A. baumannii* clinical isolates feature resistance to carbapenem antibiotics, which can greatly increase the clinical burden of treatment. In addition to intrinsic resistance, carbapenem resistance detected in these isolates is mediated by *bla*_OXA−23_. In the presence of mobile elements such as transposons, integrons and plasmids, *bla*_OXA−23_ is widely transferred among different *A. baumannii*, and this exacerbates the emergence of antibiotic resistance [2]. In addition, multidrug resistance to other antibiotics is further developed in the presence of multidrug resistance efflux pumps, outer membrane proteins and other resistance determinants. Indeed, such drug-resistant high-risk clones pose a significant threat to public health safety and require timely and effective infection control measures to prevent their spread [3].

The majority of ICU patients suffer from severe underlying diseases, have long hospital stays, and receive prolonged antibiotic therapy to minimize nosocomial bacterial infection. This can lead to their increased risk of infection with carbapenem-resistant *A. baumannii*. One study showed that highly virulent phenotypes of CRAB are a serious threat to life and health [4]. However, our understanding of such infections for patients within an ICU unit of three teaching hospitals are poorly development. In this study, we collected 92 CRAB strains isolated from the ICU of a hospital in Yangzhou, China, and assessed their genetic characteristics and transmission trajectories by whole genome sequencing, with the aim to provide a laboratory basis for the prevention and control of anbiotic resistance in this region.

## Materials and methods

### Collection of clinical A. Baumannii isolates

Samples were collected from the intensive care unit (ICU) of Yangzhou, Yixing, Xuyi hospitals from 2019 to 2022. A total of 92 *A. baumannii* isolates were cultured from sputum, blood, ascites and cerebrospinal fluid. The isolates were initially identified by VITEK mass spectrometry (BioMerieux, France).

### Antimicrobial susceptibility testing

Antimicrobial drug sensitivity to ceftazidime, cefepime, meropenem, imipenem, tigecycline, gentamicin, ciprofloxacin, and tetracycline were all assessed through a micro-broth dilution method. Disc diffusion method for resistant or apparently resistant strains is consistent with resistance results. Antimicrobial susceptibility testing results were interpreted according to CLSI-2020 [5]. *E. coli* ATCC25922 was used for quality control.

### Whole genome sequencing

Total bacterial DNA from 92 clinical CRAB isolates were extracted using a SolarbioD1600 kit (Solarbio, China). The purity of the sample was determined by NanoPhotometer^®^(IMPLEN, CA, USA), and sample concentrations determined by Qubit^®^ 3.0 Flurometer(Life Technologies, CA, USA). The clustering of the index-coded samples was performed on a cBot cluster generation system using HiSeq PE Cluster Kit v4-cBot-HS (Illumina) according to the manufacturer’s instructions. After cluster generation, the libraries were sequenced on an Illumina platform and 150 bp paired-end reads were generated.The cluster generation and sequencing were performed on Novaseq 6000 S4 platform, using NovaSeq 6000 S4 Reagent kit V1.5. Sequencing libraries were generated using Annoroad^®^ Universal DNA Fragmentase kit V2.0(AN200101-L) and Annoroad^®^ Universal DNA Library Prep Kit V2.0 (AN200101-L) according to the manufacturer’s instructions, and with index codes applied to attribute sequences to each sample. Sequencing services are provided by Annoroad Gene Tech (Beijing, China). The raw sequence data were assembled into contigs using SPAdes 3.10.0 [6].

### Genome data analysis

Acquired antimicrobial resistance genes were identified by Resfinder. VFDB was used for prediction of virulence factors. MLST was used and the bacterial genome was characterised and typed using PubMLST. (https://pubmlst.org/). Pathogenwatch was used for genome-wide species identification based on genome-wide species identification (https://pathogen.watch/). Profiles of clinical isolates of CRABs relevant to this study were uploaded to the NCBI database (Bioproject: PRJNA1002118). Graphpad and Chiplot were used for data visualization (https://www.chiplot.online/).

### Phylogenetic evolution tree construction

Snippy v4.6.0 was used to identify SNP differences in clinically isolated CRAB samples, with core.full.aln output files filtered using Gubbins v2.4.1 to obtain clean.full.aln files. Subsequently, clean.full.aln files were analyzed using Clustal Omega and a phylogenetic evolutionary tree based on core SNPs was constructed (https://www.ebi.ac.uk/Tools/msa/clustalo/). The evolutionary tree was visualized using ITOL (https://itol.embl.de/upload.cgi).

### *bla*_OXA−23_genetic environment analysis

Sequences containing *bla*_OXA−23_ in the genomes of isolates were extracted and compared using SnanpGene software (San Diego, USA). Subsequently, the Basic Local Alignment Search Tool (BLAST) was used to compare genomes uploaded through the NCBI database. MAUVE was used for gene covariance analysis and to map the environment around the *bla*_OXA−23_ gene.

## Results

### Clinical characteristics of CRAB strains

**Fig. 1:**
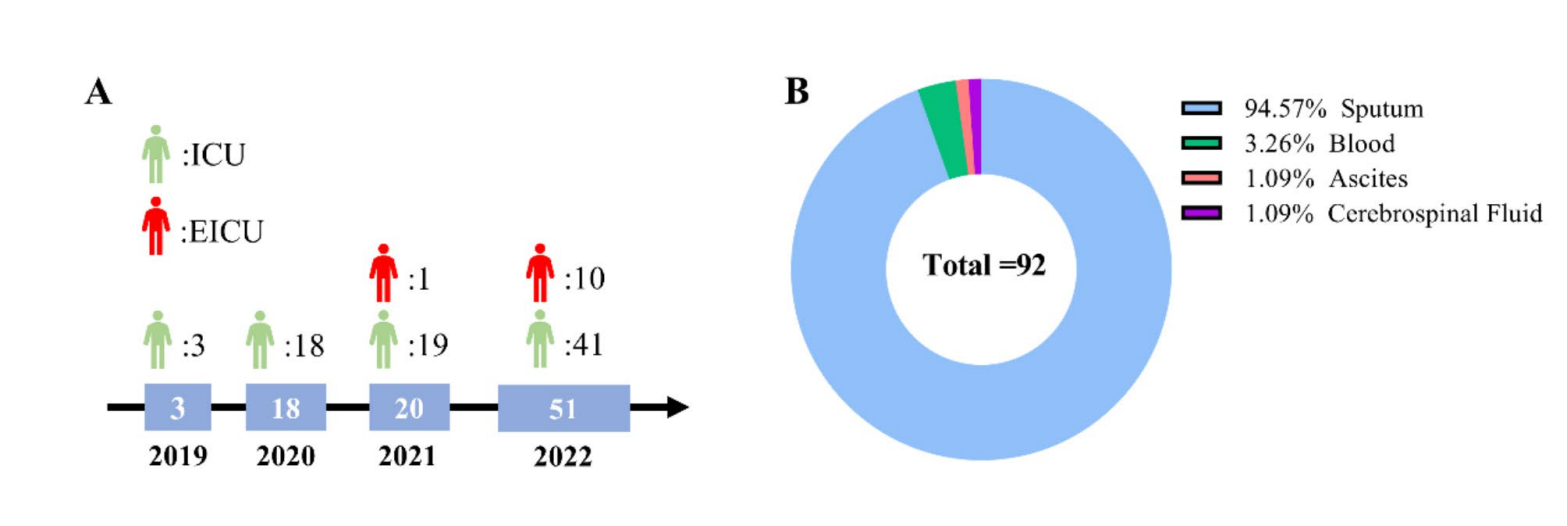
Details of annual collections of CRAB isolates and sample types. **A**: Year of isolation and number of isolates, **B**: Type of sample from which the isolates originated

A total of 92 unduplicated isolates were collected from ICU patients over a four-year period from 2019 to 2022 that were clinically documented with carbapenem-resistant *A. baumannii* infections. Isolates were predominantly of ICU origin (*n* = 81, 88%), with a small proportion of emergency ICU (EICU) origin (*n* = 11, 12%). Three CRAB samples were collected from ICUs in 2019, 18 from ICUs in 2020, as well as 19 isolates from ICUs and 1 isolate from an EICU in 2021. In 2022, 41 isolates were documented from ICU and 10 isolates were documented from EICU. Of these samples of CRAB isolates, the majority were from sputum (*n* = 87, 94.57%), with the remainder from blood (*n* = 3, 3.26%), ascites fluid (*n* = 1, 1.09%) and cerebrospinal fluid (*n* = 1, 1.09%) samples. The demographic information and clinical information of patients is shown in Fig. 1 and Table S1, respectively.

### Statistical analysis of drug resistance in CRAB subjects

Our analysis showed that all 92 CRAB strains were resistant to carbapenem antibiotics. For example, we found 100% resistance to cefepime and ceftazidime, 96% resistance to Cefepime, 91% resistance to levofloxacin, 89% resistance to Tetracycline and to Gentamicin and 83% resistance to amikacin. Of the antibiotics tested, the CRAB samples were the least (28%) resistant to minocycline. We also found that the MIC_50_ and MIC_90_ of the antibiotics tested for the CRAB samples all varied considerably. For example, for imipenem and meropenem, their MIC_50_ and MIC_90_ featured a difference of greater than 4-fold, while an 8-fold difference in amikacin was also observed. The specific statistical results are shown in Table 1, and the resistance and MIC results are shown in Table S2.

**Table 1:** Drug resistance features of clinical isolates, alongside their MIC_50,_ MIC_90_ values

Antibiotics	*R* (***n***, %)	MIC_50_	MIC_90_
Ceftazidime	92, 100%	≥ 64	≥ 64
Cefepime	88, 96%	≥ 32	≥ 64
Imipenem	92, 100%	≥ 16	≥ 64
Meropenem	92, 100%	≥ 16	≥ 64
Tetracycline	82, 89%	≥ 16	≥ 16
Minocycline	26, 28%	≥ 8	≥ 16
Gentamicin	82, 89%	> 32	> 32
Amikacin	76, 83%	≥ 8	≥ 64
Levofloxacin	84, 91%	≥ 8	≥ 16

### Analysis of bacterial genomic data

**Fig. 2:**
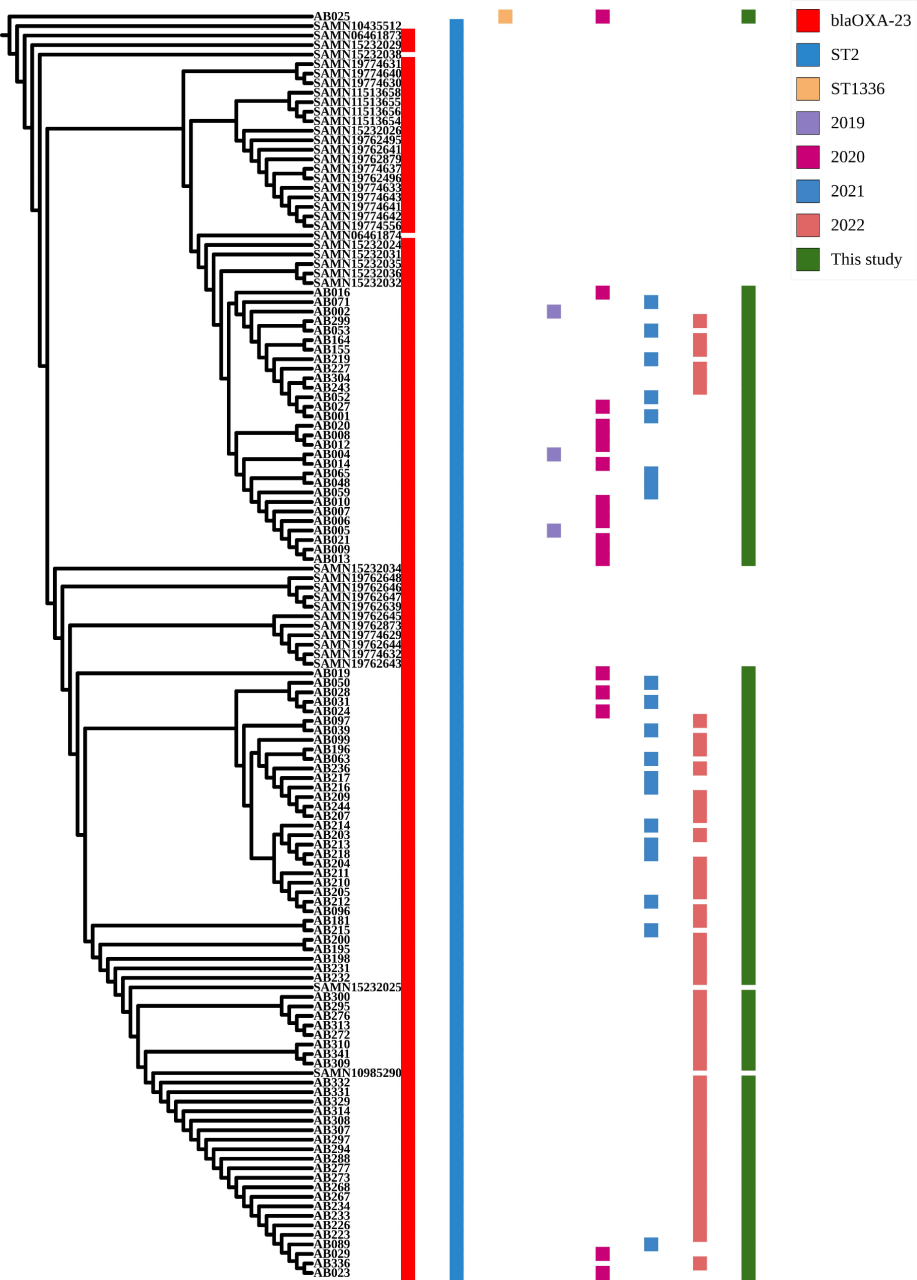
Bacterial genome evolution tree. The blaOXA-23 carriage rate, isolation time and sequence type of the isolates are shown on the right. Green represents the isolates in this study, other strains were derived from *A. baumannii* isolates from other regions of China that were publicly available in the NCBI database

When we analyzed the genomic data of the strains, we found that almost all of the strains were identified as ST2, while only one strain was identified as ST1336. In the ST2 lineage, all strains carried Class D carbapenemase *bla*_OXA−23_, and no *bla*_KPC_, *bla*_NDM_, *bla*_IMP_, or *bla*_VIM_ were found in the genome. AB25 belonging to ST1336 not carrying *bla*_OXA−23_, *bla*_KPC_, *bla*_NDM_, *bla*_IMP_, and *bla*_VIM_. Evolutionary tree analysis was performed and we identified a temporal correlation of the strains. AB25 exhibits significant differences from the ST2 lineage. In particular, the isolates in this study were closely related to isolates from Guangzhou and Hangzhou, China (Biosample: SAMN10985290 and SAMN15232025). The results of evolutionary tree and distribution of resistance genes are shown in Fig. 2.

### Bacterial genome virulence gene data analysis

**Fig. 3:**
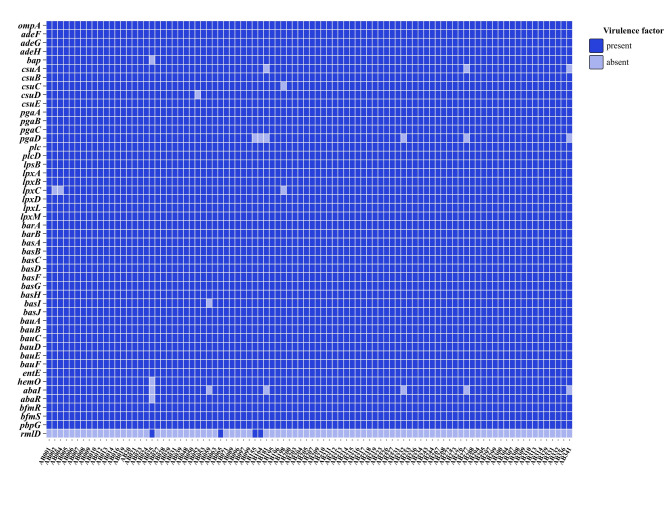
Distribution of bacterial virulence genes that are present (*dark blue*) or absent (*light blue*)

Bacterial genomes were uploaded to VFDB for virulence gene analysis. Through this, we discovered a few number of deletions in *csuA* (*n* = 3, 3.26%), *csuB* (No absent), *csuC* (*n* = 1, 1.09%), *csuD* (*n* = 1, 1.09%), and *csuE* (No absent). We detected a small deletion to *lpxC*, a cell membrane lipid A biosynthesis gene, and no Polymyxin resistance was found in combination with bacterial resistance across the samples. In addition, we also detected isolated deletions in the group sensing system *abaI* and *abaR* genes, and the rhamnose synthase gene *rmlD* was found to be largely absent. The results of the distribution of virulence genes are shown in Fig. 3.

### Single nucleotide polymorphism analysis of CRAB genome

We found that single nucleotide polymorphisms (SNPs) showed tendency to gradually disperse that was associated with the year of sample collection. For example, isolates from 2019 tended to be homogeneous, while samples from 2020 had only one dispersed isolate, however the isolates tended to be dispersed from each other. Isolates from 2021 were the most dispersed. One caveat of these observations is that the variation in the number of samples collected between years is large. Detailed results of SNP analysis are shown in Figure S1.

### Analysis of *bla*_OXA−23_gene environment

**Fig. 4:**
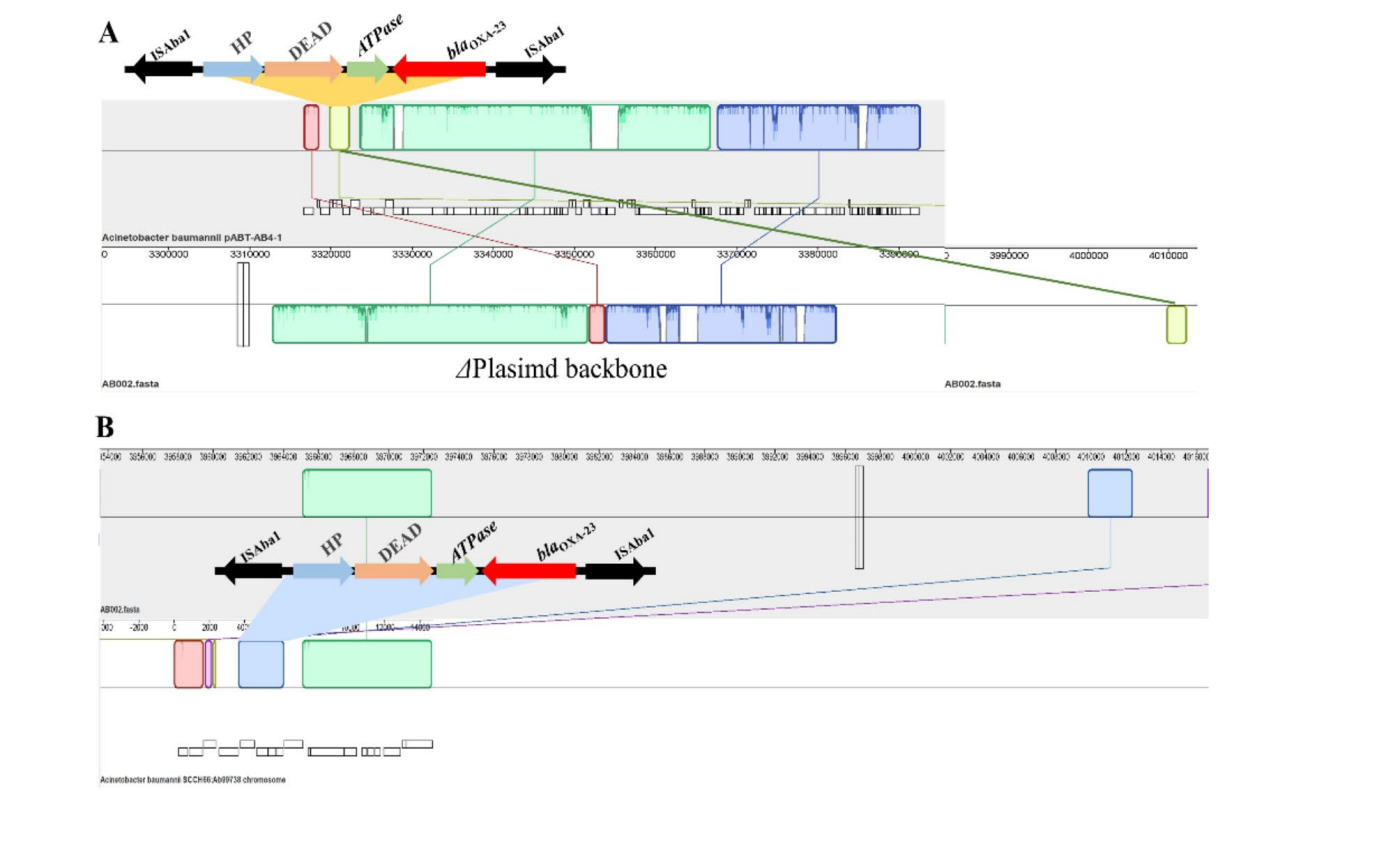
Resistance gene-gene environment analysis. **A**: plasmid homologous gene environment analysis, **B**: chromosome homologous gene environment analysis

The genome was uploaded to NCBI search and the CRAB genome used in this study was compared with the database. The *Acinetobacter baumannii* pABT-AB4-1 plasmid carries the insertion sequence *bla*_OXA−23_ in ISAba1 with homology to *bla*_OXA−23_ in the AB002 genome. In the AB002 genome, *bla*_OXA−23_ is homologous to the insertion sequences and genes as the genomes of other clinical strains in the database. This is consistent with the interpretation that *A.baumannii bla*_OXA−23_ is widely transmissible and was transmitted to these samples. The results of gene environment analysis are shown in Fig. 4.

## Discussion

The ICU patients that were documented with clinical infections in this study were associated with underlying diseases and had undergone invasive operations. Indeed, ICU patients have a higher risk of infection [7, 8]. Our data suggests that clonal transmission can occur in ICU patients even with different times of origin and site of infection, as evidenced in our phylogenetic evolutionary tree analysis. As such, our results recognize that prompt and effective infection prevention and control measures should be taken in such patients to minimize or eliminate the spread of such drug-resistant clones that will seriously affect their health.

Our whole genome sequencing data showed that the CRAB isolated in this study were all of Chinese epidemic lineage ST2, with the exception of a sample identified as AB25 (ST1336) [9, 10]. ST2 refers to a unique genetic marker obtained in a specific gene sequence that represents a particular genotype or variant. This typing is useful for epidemiological investigations, infection control and drug resistance studies, as it can help identify and track specific bacterial strains. Clonal Complex (CC) is a group of genetically related bacterial sequence types, usually consisting of one or more common sequence types that exhibit a common genetic background. CC2 is a clone with ST2 as its core and usually includes ST2 and other sequence types associated with it.CC2 is often found in hospital settings and usually exhibits multi-drug resistance, including resistance to commonly used antibiotics such as carbapenems. The spread and prevalence of CC2 clonotypes is often associated with hospital-acquired infections. Limited information is available on the ST1336 lineage, which has been identified in some hospitals in China and Vietnam [11, 12].

Identifying ST2 typing and CC2 clonotypes can help in the development of hospital infection control strategies. Knowing which bacterial strains are transmitted in hospitals helps in developing appropriate isolation measures and disinfection procedures.ST2 typing and CC2 clonotypes provide important information for studying the genetic characteristics, modes of transmission, and drug resistance of *A. baumannii*. Through these typing and clonotyping studies, healthcare facilities can more effectively implement infection control measures and optimize treatment strategies to meet the challenges posed by this multi-drug-resistant pathogen. The ST2 lineage in this study carried *bla*_oxa−23_ which we recognize to comprise the main mechanism that mediates resistance to carbapenem antibiotics in these instances, in line with previous reports [13]. Some results showed that *bla*_OXA−23_ was able to transfer rapidly in *Acinetobacter baumannii*, most likely via horizontal transmission through a plasmid form as well as a vertical integration into the chromosomal genome through the action of mobile bacterial elements [14, 15]. In addition, *armA* is a class of 16s methylases associated with high/level resistance to Gentamicin [16]. The *tet* family is also a common tetracycline resistance gene that is widely distributed among various types of bacteria [17]. As such, the MDR profile of CRAB ST2 is underpinned by the action of multiple resistance determinants [18].

*A.baumannii* biofilms have long been an important virulence factor that promotes long-term colonisation and recurrence within human hosts and of environmental sites by these pathogens. Biofilms are relevant to a variety of biological processes such as horizontal transmission of anbiotic resistance genes and immune evasion [19, 20]. Our analyses showed that the CRAB in this study carried a number of biofilm synthesis-associated genes *csuA*, *csuB*, *csuC*, *csuD*, *csuE*, *lpxC*, and we therefore hypothesised that these drug-resistant strains may be able to achieve long-term colonisation and clonal transmission in these environments through biofilm formation [21, 22]. Our results found that these drug-resistant strains show a high degree of homology in their genetic background, yet they have evolved over time. This suggests potential genetic adaptation towards greater suitability for human and healthcare environments. In particular, the isolation of CRAB originating from multiple sterile sites such as blood and cerebrospinal fluid in our study suggests that the virulence of these resistant isolates may be further enhanced. Our results emphasize the importance of continuous surveillance of these resistant strains.

There are several shortcomings in our current study. Firstly, our findings are based on samples from a single-center, and with limitations in strain collection. As such, based on our findings, we cannot establish whether cross-regional spread of the CRABs classified in this region took place or not. Secondly, although we showed the spread and evolutionary history of drug-resistant clones in our samples through phylogenetic evolutionary tree and SNP, biological trait changes involving these strains need to be confirmed by in vitro experiments. Thirdly, this study lacked data from environmental sampling, such that the prevalence of such drug-resistant clones on abiotic surfaces and in the surrounding environment could not be ascertained. Given that the majority of the strains in this study originated from respiratory samples, we must also entertain the possibility of environmental transfer of the detected infectious microorganisms from ICU or the surrounding environment.

## Conclusion

Our study has described the clinical infection characteristics, resistance profiles and genomic characteristics of ICU patients with CRAB infections detected within a hospital in Yangzhou, China. Through the application of bioinformatic tools, we tracked the epidemiological trajectory of CRAB ST2 resistance profiles in ICU patients and identified evidence for clonal transmission of these resistant clones in ICU patients. Our findings indicate that timely and effective infection control precautions should be taken to minimize or eliminate the spread of MDR microorganisms in the hospital setting.

## Electronic supplementary material

Below is the link to the electronic supplementary material.

Supplementary Material 1

Supplementary Material 2

Supplementary Material 3

## Data Availability

No datasets were generated or analysed during the current study.
