# Supplementary figures and images for: Understanding the clinical and molecular epidemiological characteristics of carbapenem-resistant Acinetobacter baumannii infections within intensive care units of three teaching hospitals
Source: Ann Clin Microbiol Antimicrob. 2025 Jan 13;24:2. doi: 10.1186/s12941-024-00766-4 (PMC11731405; doi:10.1186/s12941-024-00766-4)

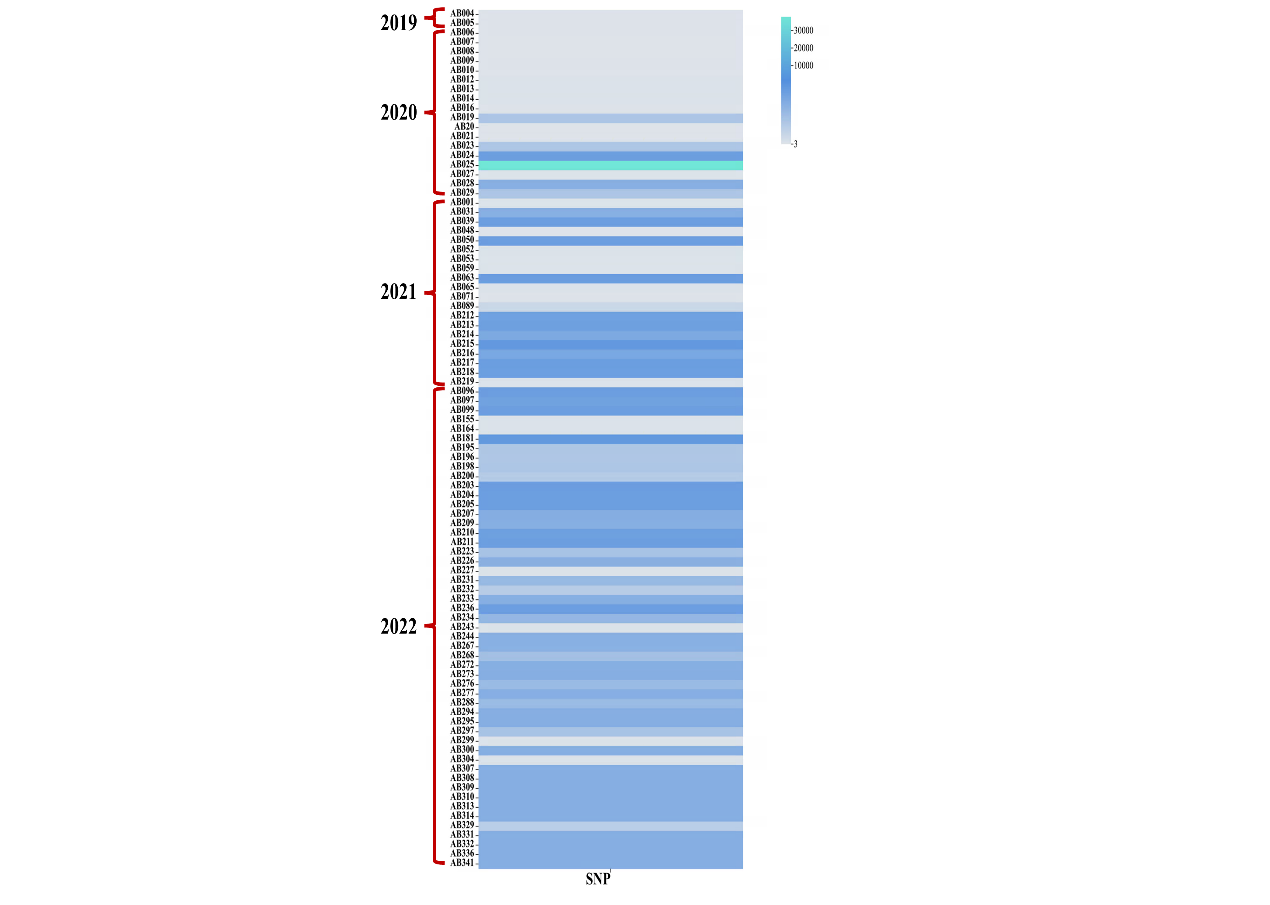

Supplement: Supplementary file 3 — Supplementary Material 3 [file 12941_2024_766_MOESM3_ESM.png]
